# Verbascoside alleviates renal fibrosis in unilateral ureteral obstruction rats by inhibiting macrophage infiltration

**DOI:** 10.22038/ijbms.2021.52759.11903

**Published:** 2021-06

**Authors:** Guihua Zhang, Fuxun Yu, Rong Dong, Jiali Yu, Meng Luo, Yan Zha

**Affiliations:** 1 Guizhou University School of Medicine, Guizhou University, Gui Yang, Gui Zhou, China; 2 The NHC Key Laboratory of Pulmonary Immunological Disease, Guizhou Provincial People’s Hospital, Gui Yang, Gui Zhou, China; 3 Department of Nephrology, Guizhou Provincial People’s Hospital, Gui Yang, Gui Zhou, China; 4 Department of Thoracic Surgery, Guizhou Provincial People’s Hospital, Gui Yang, Gui Zhou, China

**Keywords:** Fibrosis, Macrophage infiltration, Obstructive nephropathy, Verbascoside, Unilateral ureteral - obstruction

## Abstract

**Objective(s):**

To explore the effect of verbascoside on renal fibrosis in unilateral ureteral obstruction (UUO) rats.

**Materials and Methods:**

Twenty Sprague-Dawley rats were randomly distributed into sham-operated, UUO, and UUO+Verbascoside groups. After two weeks of rat model construction, urine and blood samples were collected for biochemical analysis while kidney tissues were harvested for hematoxylin and eosin (H&E), Masson’s Trichrome, and immunohistochemistry staining. Pearson coefficient was used to analyze the correlation between the two proteins.

**Results:**

Verbascoside improved UUO-induced renal dysfunction as detected by decreased serum creatinine, urea nitrogen, and urinary protein excretion rate. In UUO rats, H&E staining result revealed increased total nucleated cell number, and Masson’s Trichrome staining results showed tubular interstitial fibrosis with the deposition of collagen fibrils. Besides, expressions of fibrosis-related proteins including collagen type I (COL-I), α-smooth muscle actin (a-SMA), and tissue inhibitor of metalloproteinase 2 (TIMP2) expressed higher in the UUO group. Moreover, macrophage infiltration-related factors such as CD68+, F4/80+ cells, and suppressor of cytokine signaling-3 (SOCS3) were significantly higher in the UUO group than in sham-operated rats. However, after administration with verbascoside, the accumulation of collagen fibrils and total nucleated cell numbers were mitigated. Likewise, macrophage infiltration was extenuated and fibrosis-related proteins were down-regulated in the UUO+Verbascoside rats. Correlation analysis indicated that macrophage infiltration-related markers were related to fibrosis-related factors.

**Conclusion:**

Verbascoside could alleviate renal fibrosis in UUO rats probably through ameliorating macrophage infiltration.

## Introduction

Renal fibrosis resulting from various obstructions of the urinary tract is a common issue in long-term obstructive uropathy patients (1-4). Obstructive nephropathy caused by kidney stones accounts for 1–15% of the global general population. The prevalence in China is about 6.1%–6.4%, while it is higher in South China (5-7). Though the obstructive condition can be relieved through surgery, kidney fibrosis ca not be eliminated thoroughly (8). Therefore, effective treatment for ameliorating kidney fibrosis in obstructive nephropathy is still urgent (9-11). 

The unilateral ureteral obstruction (UUO) animal model is often used to reveal useful biomarkers and new therapy for progressive renal fibrosis. The main pathogeneses of renal fibrosis after UUO are: (a) interstitial macrophages infiltration, which produces cytokines responsible for tubular apoptosis and fibroblast proliferation and activation; (b) tubular cell death by apoptosis and necrosis leading to the formation of tubular atrophy; (c) phenotypic transition of resident renal cells (3).

Perennial infiltration of inflammatory cells including mast cells, T cells, and monocytes/macrophages, etc. is one of the vital pathways that cause renal fibrosis by providing the fibrotic microenvironment (4, 12-14). Thereinto, macrophage infiltration makes a great difference through two aspects. On the one hand, profibrotic cytokines and growth factors released from macrophages during the inflammation process are capable of exacerbating renal fibrosis (15, 16). Macrophages produce C-X-C chemokine receptor 3 and monocyte chemoattractant protein 1 which promotes myofibroblasts to generate extracellular matrix proteins and participate in renal fibrosis subsequently (15). Research demonstrated that macrophage-specific Krüppel-like factor 4 deletion could exacerbate kidney fibrosis with increased levels of collagen I (COL-I) and a-smooth muscle actin (a-SMA) in the injured kidney (17). On the other hand, macrophage to myofibroblast transition (MMT) plays a key role in renal fibrosis (18). These cells increased in the tubulointerstitial areas of UUO kidneys contribute to progressive renal fibrosis with ~50% efficiency of cell transformation (3, 12, 19). A study provided evidence that macrophages, predominantly M2 phenotype, could directly transdifferentiate into collagen-producing myofibroblasts, resulting in renal fibrosis (18). Research verified that the MMT process in the development of interstitial fibrosis was mediated by transforming growth factor-β/small mothers against decapentaplegic 3 (TGF-β/Smad3) signaling (19). On a more cautious note, findings show that suppressor of cytokine signaling-3 (SOCS3), a key protein for mediating the activity of inflammatory cytokines in macrophage infiltration proceedings, expressed in both cytoplasm and nucleus of primary human macrophages and SOCS3 protein was more up-regulated in acute, chronically and granulomatously inflamed human tissues than in corresponding normal tissues (20-23). 

Previous studies have reported that proliferation of interstitial fibroblasts derived from macrophage transformation and non-transformed fibroblasts such as myofibroblast may lead to abundant deposition of the extracellular matrix and renal fibrosis (3, 24, 25), and the accumulation of fibrotic markers, such as COL-I and a-SMA are the main locally pathological features in experimental animal models and patients with obstructive nephropathy (4). Alpha-SMA, a marker of activated fibroblasts, was up-regulated in fibrosis and accumulation of COL-I increased in renal interstitium (26). Reports have also shown that tissue inhibitors of metalloproteinases (TIMPs) like TIMP2 played an important role in maintaining the inner balance of the extracellular matrix (27). 

Verbascoside (a kind of phenylethanoid glycoside, also known as acteoside or kusagin) is widely used in traditional Chinese medicine and can be found in many medicinal plant families such as *Verbenaceae *and* Plantago* species (28-31). Recently, interest in verbascoside has been growing due to its multiple biological activities including antioxidant, anti-inflammatory, neuroprotection, muscle atrophy relieving, and wound healing along with anti-proliferative effects in cancer (32). Researchers found that verbascoside was a weak inhibitor for many cytochrome P (CYP) enzymes which could be considered as an adjuvant during tuberculosis treatment (33). Paola* et al.* demonstrated that verbascoside could exert an anti-inflammatory effect in experimental periodontitis and ameliorate the tissue damage caused by ligation periodontitis (32). It also has been reported that verbascoside possesses potent anti-osteoporotic properties (34). Remarkably, verbascoside might be of benefit in suppressing the development of fibrosis in the liver by modulating pro-inflammatory cytokine production (35).

However, whether verbascoside can mitigate the degree of renal fibrosis is still not clear. Thus, the purpose of this study is to elucidate the effect of verbascoside on renal fibrosis and explore the underlying mechanism of verbascoside affecting the kidney fibrosis of obstructive nephropathy.

## Materials and Methods

***Animals***

Twenty 6-8 week old, approximately 200 g pathogen-free male Sprague-Dawley (SD) rats were purchased from Chongqing Teng Xin Biotechnology Co., Ltd. (Chongqing, China). The rats were housed in the standard temperature (21 °C ±2 °C) and standard humidity (55%±2%) with regular 12:12-hr light/dark cycles and allowed to have free access to standard laboratory food and water. 

***Experimental design***

**Figure 1:**
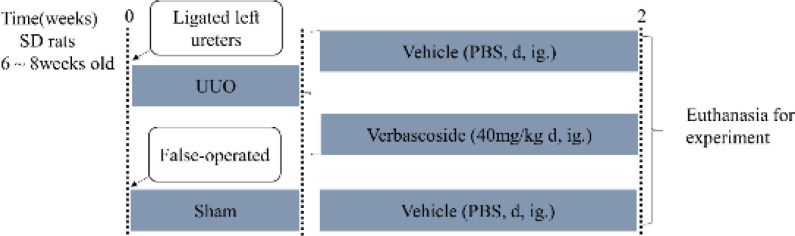
Research design of the rat experiment. The UUO model was established by ligating the rats’ left ureters with 4-0 silk thread at time 0. n = 6 or 7 rats in each group. After the postoperative day of UUO, the rats were treated with verbascoside (40 mg·kg-1 body weight per day, ig) for 2 weeks. The sham rats and the UUO rats received solvent (normal saline, pH 7.3, 0.154 mol·L-1). Age-matched false-operated rats were used as controls

UUO is a well-established experimental rodent model that simulates the pathogenesis of obstructive nephropathy in humans (3, 36-38). So, the UUO model causing progressive renal fibrosis in rodents was used in this study. All rats that underwent UUO surgery were performed as previously described (3, 39, 40). The animals were randomly divided into three groups: (a) the sham-operated group in which rats underwent the same procedure without the ligation of one ureter; (b) the UUO group in which the rats’ left ureters were ligated with 4-0 silk thread; (c) the treatment group in which rats received intragastrical gavage of verbascoside (40 mg·kg^-1^ body weight per day (31, 41, 42), solubilized in normal saline, pH 7.3, 0.154 mol·L^-1^) for 14 consecutive days after the postoperative day of UUO (Figure 1, n = 6 or 7 rats per group). Rats from the sham-operation and UUO group received normal saline. After the operation, all animals were recovered in a heated environment with an intelligent temperature metal bath and returned to their cages. Two weeks following surgery or treatment, each rat was placed in a metabolic cage one day in advance to collect 24 hr urine before euthanizing with an intraperitoneal injection of 1% chloral hydrate solution, blood collected, and tissues perfused using saline with heparin. The kidneys were harvested and fixed in formalin fixative with 10% phosphate buffer (Babio Biotechnology Co., Ltd., Jinan, China) bought for histological analysis.

***Biochemical examination***

The urinary protein excretion rate was detected using the 24-hr urine collected the day before sacrificing with a protein estimation kit (BioAssay Systems, Hayward, USA), and the serum separated from whole blood samples by cardiac puncture at the time of sacrifice was used to determine blood urea nitrogen and serum creatinine concentration using the urea assay kit and the creatinine assay kit, respectively (BioAssay Systems, Hayward, USA). Three biochemical indexes were calculated according to the manufacturer’s protocols.

***Histopathological staining***

Renal tissues fixed in formalin fixative were embedded in paraffin and sliced into 4 μm thickness. Deparaffinizing, rehydrating, and staining kidney sections by hematoxylin and eosin (H&E, Boster Biological Technology Co.Ltd, Wuhan, China) and Masson’s Trichrome (Sigma-Aldrich, Merck KGaA, Darmstadt, Germany) following proposed protocols. Thereinto, the number of the total nucleated cell was counted by H&E staining. Then the degree of renal fibrosis was evaluated based on the amount of collagen deposition (blue color area over the whole cortex area by Masson’s Trichrome staining) in the renal interstitial regions with Masson’s Trichrome staining and expressed as a percentage of renal interstitial proportion relative to the entire area using a computerized image analysis system (Image-Pro Plus v 6.0). From each kidney, ten random interstitial cortical pictures were captured at 400magnification using a high-resolution video camera (Leica, Germany) connected to a light microscope (Leica DM 300 LED).

***Immunohistochemistry***

Portions of kidney paraffin sections (4 μm thickness) were dewaxed in xylene and rehydrated in alcohol gradient. Then they were blocked in 3% [w/v] hydrogen peroxide (H_2_O_2_) for 10 min at room temperature to eliminate endogenous enzymes. After boiling in antigen retrieval solution for 15 min in a high-pressure cooker, the sections were incubated overnight at 4 °C with appropriate primary antibodies [α-SMA, COL-I, TIMP2, F4/80^+^, CD68^+^(1:100 dilution), and SOCS3(1:200 dilution); Abcam, Cambridge, UK]. The antibody was diluted using dilution [0.01 mol/l phosphate-buffered saline (PBS) containing 1%[w/v] bovine serum albumin (BSA)]. After washing with PBS, the secondary antibody [rabbit anti-mouse immunoglobulin (Ig) G (1:200 dilution) for F4/80^+^, CD68^+^ and goat anti-rabbit IgG (1:200 dilution) for α-SMA, COL-I, TIMP2, and SOCS3 proteins; Abcam, Cambridge, UK] was applied for 2 hr at room temperature and incubated for 30 min in 37 °C thermostat-controlled water-bath. Finally, a 3, 3’-diaminobenzidine (DAB) kit (Beijing Zhongshan Golden Bridge Biotechnology Co.Ltd., Beijing, China) was used to visualize the signal. The sections were then counterstained with hematoxylin, dehydrated, and mounted with the mounting medium. Ten images of each kidney at 400magnification were taken under a Leica microscope. Image-Pro Plus image 6.0 analysis software was used to calculate the protein levels of α-SMA, COL-I, TIMP2, SOCS3, and then expressed as mean density for these proteins (integral optical density (IOD)/area). F4/80^+ ^and CD68^+^ cell numbers were counted. The principle of randomized double-blind control was followed to IHC image acquisition and statistical analysis.

***Statistical analysis***

All values were expressed as mean ± standard error (SEM). Shapiro-Wilk normality test was applied to test data distribution normality with statistical product and service solutions (SPSS) software (version 22.0, IBM Corp, Armonk, NY, USA). For comparisons between groups with normally distributed data, statistically significant differences were determined by using an unpaired two-tailed Student’s t-test for categorical variable comparisons with GraphPad Prism 6.0 package (GraphPad Software, San Diego, CA, USA). The Mann-Whitney test was used for comparisons between groups with non-normally distributed variables. Pearson coefficient was used to analyze the correlation between two variables. *P*-value<0.05 was considered statistically significant. The UUO group was compared with the sham group and the UUO + Verbascoside (Verb) group was compared with the UUO group when conducting the differential analysis of pathological staining and immunohistochemistry experiments.

## Results

***Verbascoside improved UUO-induced renal dysfunction***

**Figure 2:**
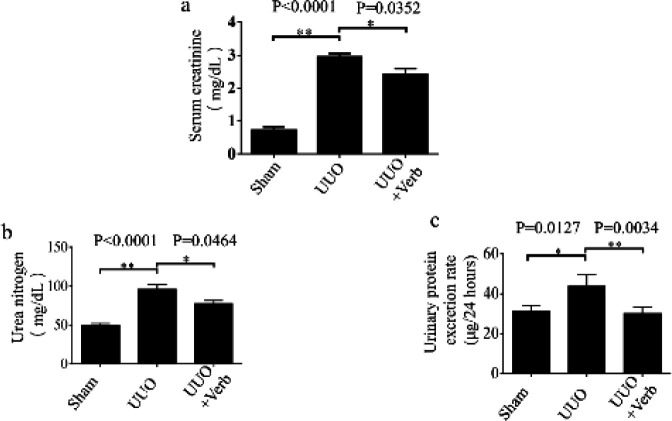
Renal function-related biochemistry indexes in each group of rats. (a) Serum creatinine level. (b) Urea nitrogen level, and (c) Urinary protein excretion rate. The UUO model was established by ligating the rats’ left ureters with 4-0 silk thread, and on the postoperative day of UUO, animals were administered with verbascoside by intragastrical gavage for two weeks. Values are represented as means ± standard error (SD); n=6 or 7 rats per group. Mann-Whitney U test was used for two-group comparisons of Serum creatinine, and two-tailed student’sStudent’s t-test was used for single two-sample comparisons of Ureaurea nitrogen and Urinaryurinary protein excretion raterates. Verb: verbascoside. *P<0.05; **P<0.01

To evaluate the efficacy of verbascoside in renal fibrosis, the experimental UUO model was used in this study, and verbascoside was given after ligaturing the left ureter. Then serum creatinine, urea nitrogen, and urinary protein excretion rate were detected to assess the rats’ kidney function changes. Compared with the sham-operated group, serum creatinine, urea nitrogen, and urinary protein excretion rates were higher in the UUO group (*P<*0.0001, *P<*0.0001, and *P=*0.0127, respectively in Figure 2). However, the effect could be significantly relieved with verbascoside treatment (*P=*0.0352, *P=*0.0464, and* P=*0.0034, respectively, Figure 2).

***Verbascoside modified UUO-induced renal pathological alterations***

**Figure 3:**
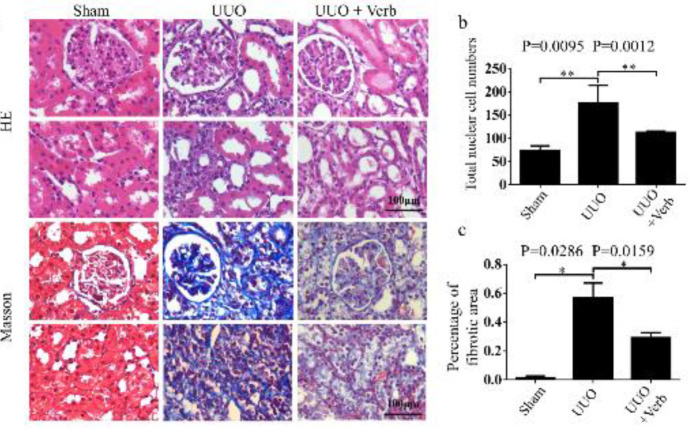
Renal histopathology change in all groups. (a) Paraffin sections of kidney tissues from sham/UUO model/ UUO + Verb rats were stained with H&E and Masson's trichrome. Original magnification, 400×. Scale bars, 100 μm. (b) Bar graph of the total nuclear cell calculated by HE staining. (c) Percentage of the fibrotic area by Masson's trichrome staining. All values are presented as SEM. n=6 or 7 rats in each group. Mann-Whitney U test was used for single two-sample comparisons. Verb: verbascoside. *P<0.05;, **P<0.01

To verify whether the therapeutic effect of verbascoside on renal pathological changes was in accordance with biochemical examination results, pathological staining of kidney tissue was performed. H&E staining indicated renal pathological alterations such as tubular dilatation, and loss of brush border in UUO rats was alleviated after treatment with verbascoside (Figure 3a). Moreover, Masson’s trichrome staining of kidney tissues showed that UUO surgery significantly increased the deposition of collagen fibrils compared with sham-operated rats (Figures 3a and c, *P=*0.0095). Then with verbascoside treatment, accumulation of collagen fibrils (semi-quantitative as percentage of the fibrotic area) was reduced (Figures 3a and c, *P=*0.0159). In addition, the total nucleated cells highly expressed in UUO rats (Figure 3b,* P=*0.0286), which meant unconventional cell appearance in the kidney tissue was reduced after treating with verbascoside (Figure 3b,* P=*0.0012). 

***Verbascoside ameliorated macrophage infiltration in the kidney***

**Figure 4:**
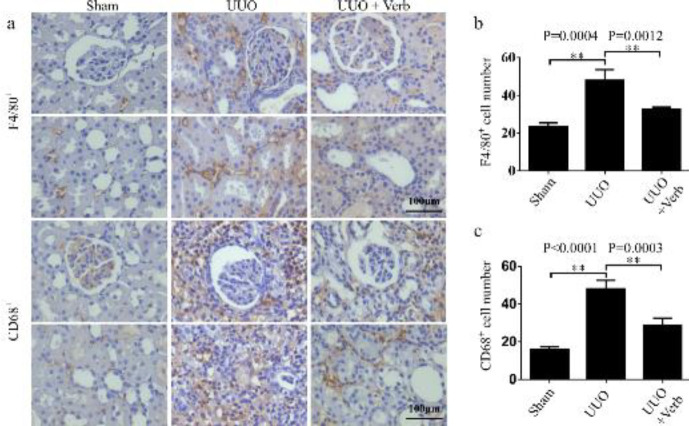
Macrophage infiltration levels in the kidney of different experimental groups. (a) Expression of F4/80+ and CD68+ cells by immunohistochemistry. Original magnification, 400×. Scale bars, 100 μm. (b) Bar graph of F4/80+ cell count calculated by immunohistochemistry. (c) Bar graph of CD68+ cell count. All values are presented as SEM. n=6 or 7 rats per group. Two-tailed student’sStudent’s t-test was used for single two-sample comparisons. Verb: verbascoside. *P<0.05; **P<0.01

Macrophages are some of the main infiltration cells in the process of inflammation progress and renal fibrosis (43, 44). The assumption was that infiltration of macrophages might be related to the augment of the total nucleated cells. Therein, typical macrophages (F4/80^+ ^and CD68^+^ positive cells) were tested in this study to verify the hypothesis and to evaluate the effects of verbascoside on renal macrophage infiltration in UUO rats. As shown in the immunohistochemical images, typical macrophages were found in the tubulointerstitium which was consistent with the hypothesis (Figure 4a). The expressions of F4/80^+^ and CD68^+^ positive cells in UUO rats were significantly higher compared with the sham group (*P=*0.0004 and *P<*0.0001, respectively, Figures 4a-c). While after two weeks of treatment with verbascoside, the numbers of F4/80^+^ and CD68^+^ positive cells in the kidney decreased significantly (*P=*0.0012 and* P=*0.0003, respectively, Figure 4). 

***Verbascoside attenuated renal fibrosis in UUO rats***

**Figure 5:**
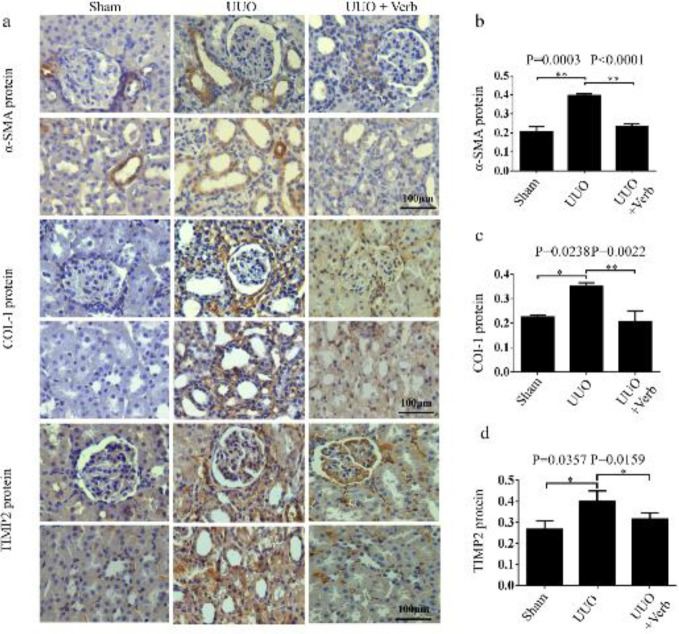
α-SMA, COL-I, and TIMP2 protein levels in the kidney of different experimental groups. (a) Expression of α-SMA, COL-I, and TIMP2 protein by immunohistochemistry. Original magnification, 400×. Scale bars, 100 μm. (b) Bar graph of α-SMA protein expression level. (c) Bar graph of COL-I protein expression level. (d) Bar graph of TIMP2 protein expression level. All values are presented as SEM. n=6 or 7 rats per group. Two-tailed student’sStudent’s t-test was used for single two-sample comparisons of α-SMA protein and the Mann-Whitney U test was used for two-group comparisons of COL-I and TIMP2 proteins. Verb: verbascoside. *P<0.05;, **P<0.01

To detect the fibrosis changes in UUO rats, the expression levels of fibrotic proteins (α-SMA, COL-I, and TIMP2) were determined using immunohistochemical staining. Results showed that relatively high-intensity staining of α-SMA, COL-I, and TIMP2 was observed in UUO rats, in comparison with the sham-operated rats (Figure 5a). And the calculated data also showed the expressions of these proteins up-regulated significantly (*P=*0.0003, *P=*0.0238, and *P=*0.0357, respectively, Figures 5b-d). After treatment, these three protein expressions were reduced (*P<*0.0001, *P=*0.0022, and* P=*0.0159, Figure 5).

Macrophage infiltration was positively related to fibrotic proteins.

**Figure 6:**
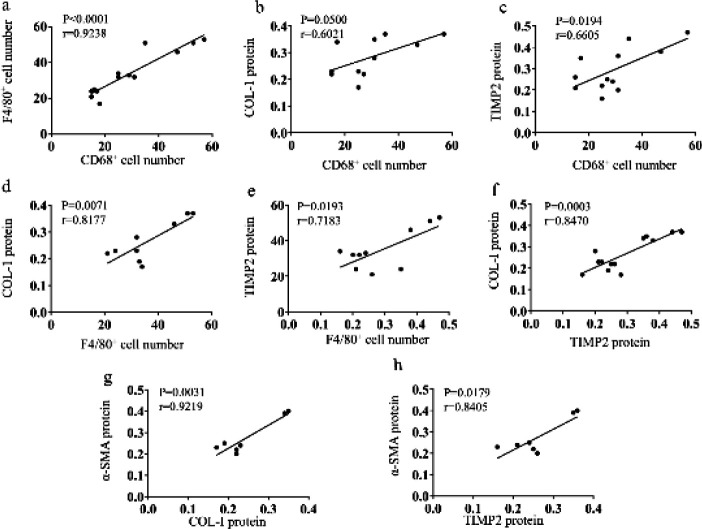
Correlation analysis of macrophage markers and fibrotic proteins. (a) CD68+ cells had a positive relation with F4/80+ cells. (b) CD68+ cell number was positively associated with COL-I protein. (c) CD68+ cell number was positively related to TIMP2 protein. (d) F4/80+ cell number was positively associated with COL-I protein. (e) F4/80+ cell number had a positive relation with TIMP2 protein. (f) TIMP2 protein and COL-I protein had a positive relation. (g) COL-I protein was positively associated with a-SMA protein. (h) TIMP2 protein had a positive correlation with a-SMA protein

Pearson’ correlation analysis was used to assess the associations among the fibrotic-related proteins or positive stain cells. It was found that macrophage-related markers (F4/80^+ ^and CD68^+^) positively correlated with two fibrotic proteins (COL-I, TIMP2), respectively (*P=*0.0071, r=0.8177, n=9; *P=*0.0193, r=0.7183, n=10; *P=*0.0500, r=0.6021, n=11 and *P=*0.0194, r=0.6605, n=12, respectively, Figures 6b-e). Furthermore, F4/80^+ ^and CD68^+^ positive cells were correlated with each other (*P<*0.0001, r=0.9238, n13, Figure 6a) yet COL-I and TIMP2 were also correlative (*P=*0.0003, r=0.8470, n=13, Figure 6f). And the expression of α-SMA protein was related to COL-I and TIMP2 proteins (*P=*0.0031, r=0.9219, n=7 and *P=*0.0179, r=0.8405, n=7, respectively, Figures 6g, h). 

***Verbascoside reduced the expression of SOCS3 protein***

**Figure 7:**
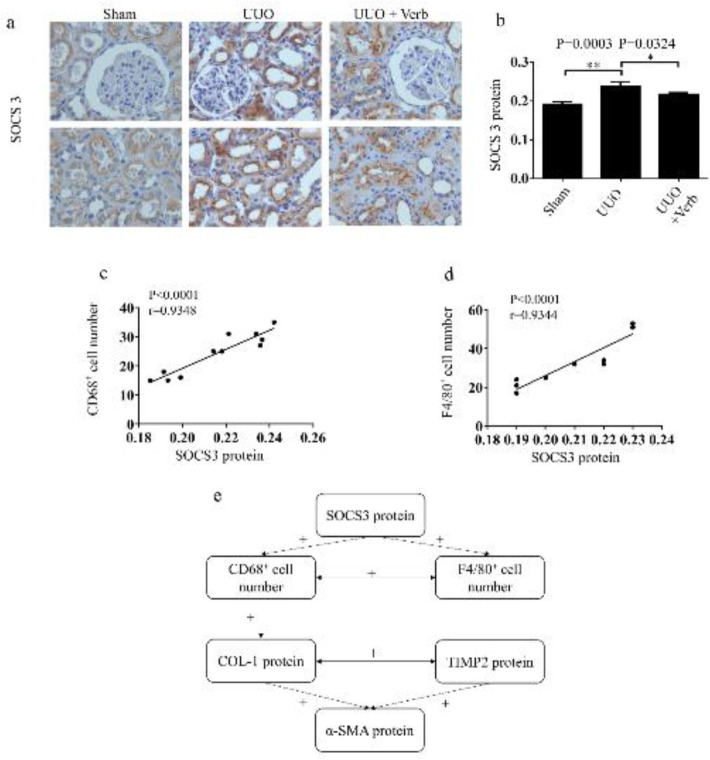
SOCS3 protein expression level in the kidneykidneys of different experimental groups. (a) Expression of SOCS3 protein by immunohistochemistry. Original magnification, 400×. Scale bars, 100 μm. (b) Bar graph of SOCS3 protein expression level. (c) SOCS3 protein was positively related to CD68+ cell number. (d) SOCS3 protein was positively associated with F4/80+ cell number. (e) The network of Correlationcorrelation analysis. +: a positive correlation. All values are presented as means±SD. n=6 or 7 rats in each group. Two-tailed student’sStudent’s t-test was used for single two-sample comparisons. Verb: verbascoside. *P<0.05;, **P<0.01

Findings show that SOCS3 is a key protein for reducing the activity of inflammatory cytokines in macrophage infiltration proceedings (19-22, 45). Therefore, to investigate whether SOCS3 participated in the process of renal fibrosis in UUO rats with macrophage infiltration, SOCS3 protein was detected using immunohistochemistry. The results showed that the expression level of SOCS3 protein was significantly increased in the UUO group compared with the sham-operation group while the up-regulation was reduced after verbascoside treatment (*P=*0.0003 and *P=*0.0324, respectively, Figures 7a, b). SOCS3 protein level positively correlated with F4/80^+^ and CD68^+^ positive cell count (*P<*0.0001, r=0.9344, n=10 and *P<*0.0001, r=0.9348, n=11, respectively, Figures 7c, d) while there was no correlation between SOCS3 and fibrosis-related proteins (α-SMA, COL-I, TIMP2) directly. 

## Discussion

Renal fibrosis resulting from obstruction of the urinary tract is a common issue in obstructive nephropathy patients (2, 46, 47). Major cellular events of renal fibrosis include: (a) inflammatory cell infiltration, (b) activation and expansion of fibroblasts from diversified sources, (c) production and deposition of large amounts of extracellular matrix (ECM) components (48). In the present study, we found that expression of COL-I, a-SMA, and TIMP2 proteins, and numbers of nucleated cells deposited in the kidney of UUO rats by comparison with the sham-operated rats increased. Moreover, H&E staining indicated renal pathological alterations such as tubular dilatation and loss of brush border in UUO rats, and Masson’s trichrome staining of kidney tissues showed that UUO surgery significantly increased the deposition of collagen fibrils. These results were consistent with the previous study (16). 

Some Chinese herbal medicines could ameliorate the pathological changes mentioned above, though effective treatment has not been confirmed. *Wang et al.* provided evidence that *Cryptotanshinone*, a bioactive constituent isolated from the Chinese herb *Salvia miltiorrhiza*, could ameliorate kidney fibrosis by improving tubular expansion and infiltrated inflammatory cells by inhibiting the TGF-β1/Smad3/integrin β1 signaling pathway (49). Hosseinian *et al.* described that Thymoquinone, a biologically active component of *Nigella sativa *Linn could alleviate renal interstitial fibrosis in rats with UUO (36). A study explained that petchiether A, a novel small-molecule meroterpenoid from *Ganoderma*, could attenuate obstructive nephropathy by suppressing TGF-β/Smad3 and nuclear factor kappa-B (NF-κB) signaling (4). Yet so far, there is no report about the effect of verbascoside on renal fibrosis.

Verbascoside is one of the most well-characterized phenylethanoid glycosides (50, 51). It has been reported that verbascoside had effects like scavenging biological free radicals, metal chelating activities, cell protection of oxidants, inhibiting lipid peroxidation, and enhancing endogenous antioxidant defense (52). Researchers proved that glioblastoma cell proliferation, glioblastoma tumor volume, and growth were decreased in a xenograft mouse model treated with verbascoside (53). A study demonstrated that verbascoside had potential therapeutic value for Parkinson’s disease by activating the nuclear factor erythroid 2-related factor/anti-oxidation reaction element (Nrf2/ARE) signaling pathway (54). Most importantly, a study verified that verbascoside had the functions of suppressing the expression of TGF-β1 in the development of hepatic fibrosis and played a role in hepatoprotective consequently (35). In this study, we found that after treating the UUO rats with verbascoside for fourteen continuous days, these kidney pathological alternations such as tubular dilatation, loss of brush border, and increase of total nucleated cells in the model rats were relieved, which implied that verbascoside was capable of modifying renal pathology changes resulting from UUO. Furthermore, the biomedical examination results indicated that verbascoside might ameliorate the renal dysfunction resulting from UUO. After analyzing the expression levels of the fibrosis-related proteins including COL-I, a-SMA, and TIMP2 in the kidney tissues, we found that these proteins’ expression levels were up-regulated in the UUO group while up-regulation was relieved in the verbascoside treatment group which indicated that renal fibrosis resulting from UUO could be attenuated by verbascoside treatment.

Macrophage infiltration in the kidneys has been considered to play a pivotal role in the development of interstitial fibrosis induced by UUO (4, 43, 55). A study suggested that verbascoside could interfere with progressive glomerulonephritis through suppression of the leukocyte accumulation in the glomeruli in rats (56). The results of F4/80^+ ^and CD68^+ ^positive cells detected in this study demonstrated that both typical macrophage markers expressed higher in model rats while they were relieved after verbascoside treatment, suggesting that verbascoside was capable of ameliorating macrophage infiltration significantly. More importantly, the correlation analysis result indicated that the infiltration of F4/80^+ ^and CD68^+ ^macrophages positively correlated with fibrotic proteins. Therefore, macrophage infiltration was likely to play a key role in kidney fibrosis.

Of particular note, findings show that SOCS3, a well-known feedback marker of the Janus kinase/signal transducers and activators of transcription (JAK/STAT) signaling pathway, mediates the signal transduction of many cytokines, and Pedroso* et al. *proved that SOCS3 played a significant role in regulating the activity of inflammatory cytokines during the infiltration of macrophages (22, 45, 57, 58). By detecting the expression of SOCS3 protein, we found that compared with the sham-operated rats, SOCS3 protein was up-regulated in UUO rats while the up-regulation was relieved after treatment with verbascoside. Correlation analysis showed that SOCS3 level was positively related to F4/80^+ ^or CD68^+ ^positive cell count. These results demonstrated that SOCS3 protein could directly mediate the process of macrophage infiltration, although how the SOCS3 protein regulates macrophage infiltration needs to be investigated.

## Conclusion

In this study, we found that verbascoside could improve UUO-induced renal dysfunction by relieving serum creatinine, urea nitrogen, and urinary protein excretion rates. Besides, verbascoside could ameliorate macrophage infiltration and alleviate the degree of renal fibrosis consequently. These results suggested that verbascoside would be a promising therapeutic drug candidate for obstructive nephropathy resulting from renal fibrosis.

## Availability of Data and Materialss

The data analyzed for this study are available from the corresponding author upon reasonable request.

## References

[1] Zhang ZH, He JQ, Qin WW, Zhao YY, Tan NH (2018). Biomarkers of obstructive nephropathy using a metabolomics approach in rat. Chem Biol Interact.

[2] Cai K, Chai L, Luo Q, Dai Z, Wu L, Hong Y (2019). Full age spectrum equation versus CKD-EPI and MDRD equations to estimate glomerular filtration rate in adults with obstructive nephropathy. J Int Med Res.

[3] Martínez-Klimova E, Aparicio-Trejo OE, Tapia E, Pedraza-Chaverri J (2019). Unilateral ureteral obstruction as a model to investigate fibrosis-attenuating treatments. Biomolecules.

[4] You YK, Luo Q, Wu WF, Zhang JJ, Zhu HJ, Lao L (2019). Petchiether A attenuates obstructive nephropathy by suppressing TGF-beta/Smad3 and NF-kappaB signalling. J Cell Mol Med.

[5] Zeng G, Mai Z, Xia S, Wang Z, Zhang K, Wang L (2017). Prevalence of kidney stones in China: An ultrasonography based cross-sectional study. BJU Int.

[6] Wu J, Yang Z, Wei J, Zeng C, Wang Y, Yang T (2020). Association between serum magnesium and the prevalence of kidney stones: a cross-sectional study. Biol Trace Elem Res.

[7] Yang C, Wang H, Zhao X, Matsushita K, Coresh J, Zhang L (2020). CKD in China: Evolving spectrum and public health implications. Am J Kidney Dis.

[8] Stevens S (2018). Obstructive Kidney Disease. Nurs Clin North Am.

[9] Waasdorp M, Rooij DM, Florquin S, Duitman J, Spek CA (2019). Protease-activated receptor-1 contributes to renal injury and interstitial fibrosis during chronic obstructive nephropathy. J Cell Mol Med.

[10] Zhao J, Meng M, Zhang J, Li L, Zhu X, Zhang L (2019). Astaxanthin ameliorates renal interstitial fibrosis and peritubular capillary rarefaction in unilateral ureteral obstruction. Mol Med Rep.

[11] Xianyuan L, Wei Z, Yaqian D, Dan Z, Xueli T, Zhanglu D (2019). Anti-renal fibrosis effect of asperulosidic acid via TGF-β1/smad2/smad3 and NF-κB signaling pathways in a rat model of unilateral ureteral obstruction. Phytomedicine.

[12] Yang L, Yuan H, Yu Y, Yu N, Ling L, Niu J (2019). Epidermal growth factor receptor mimotope alleviates renal fibrosis in murine unilateral ureteral obstruction model. Clin Immunol.

[13] Kamata M, Amano H, Ito Y, Fujita T, Otaka F, Hosono K (2019). Role of the high-affinity leukotriene B4 receptor signaling in fibrosis after unilateral ureteral obstruction in mice. PloS one.

[14] Han H, Zhu J, Wang Y, Zhu Z, Chen Y, Lu L (2017). Renal recruitment of B lymphocytes exacerbates tubulointerstitial fibrosis by promoting monocyte mobilization and infiltration after unilateral ureteral obstruction. J Pathol.

[15] Ma W, Tao L, Wang X, Liu Q, Zhang W, Li Q (2016). Sorafenib inhibits renal fibrosis induced by unilateral ureteral obstruction via inhibition of macrophage infiltration. Cell Physiol Biochem.

[16] Wang D, Xiong M, Chen C, Du L, Liu Z, Shi Y (2018). Legumain, an asparaginyl endopeptidase, mediates the effect of M2 macrophages on attenuating renal interstitial fibrosis in obstructive nephropathy. Kidney Int.

[17] Wen Y, Lu X, Ren J, Privratsky JR, Yang B, Rudemiller NP (2019). KLF4 in macrophages attenuates tnfalpha-mediated kidney injury and fibrosis. J Am Soc Nephrol.

[18] Meng XM, Wang S, Huang XR, Yang C, Xiao J, Zhang Y (2016). Inflammatory macrophages can transdifferentiate into myofibroblasts during renal fibrosis. Cell Death Dis.

[19] Wang YY, Jiang H, Pan J, Huang XR, Wang YC, Huang HF (2017). Macrophage-to-myofibroblast transition contributes to interstitial fibrosis in chronic renal allograft injury. J Am Soc Nephrol.

[20] Yin Y, Liu W, Dai Y (2015). SOCS3 and its role in associated diseases. Hum Immunol.

[21] Gao Y, Zhao H, Wang P, Wang J, Zou L (2018). The roles of SOCS3 and STAT3 in bacterial infection and inflammatory diseases. Scand J Immunol.

[22] Pedroso JAB, Ramos-Lobo AM, Donato J (2018). SOCS3 as a future target to treat metabolic disorders. Hormones (Athens).

[23] White GE, Cotterill A, Addley MR, Soilleux EJ, Greaves DR (2011). Suppressor of cytokine signalling protein SOCS3 expression is increased at sites of acute and chronic inflammation. J Mol Histol.

[24] Li S, Yu L, He A, Liu Q (2019). Klotho inhibits unilateral ureteral obstruction-induced endothelial-to-mesenchymal transition via TGF-beta1/smad2/snail1 signaling in mice. Front Pharmacol.

[25] Yao L, Wright MF, Farmer BC, Peterson LS, Khan AM, Zhong J (2019). Fibroblast-specific plasminogen activator inhibitor-1 depletion ameliorates renal interstitial fibrosis after unilateral ureteral obstruction. Nephrol Dial Transplant.

[26] Colon S, Luan H, Liu Y, Meyer C, Gewin L, Bhave G (2018). Peroxidasin and eosinophil peroxidase, but not myeloperoxidase, contribute to renal fibrosis in the murine unilateral ureteral obstruction model. Am J Physiol Renal Physiol.

[27] Bhuvarahamurthy V, Kristiansen GO, Johannsen M, Loening SA, Schnorr D, Jung K (2006). In situ gene expression and localization of metalloproteinases MMP1, MMP2, MMP3, MMP9, and their inhibitors TIMP1 and TIMP2 in human renal cell carcinoma. Oncol Rep.

[28] Reid AM, Juvonen R, Huuskonen P, Lehtonen M, Pasanen M, Lall N (2019). In Vitro human metabolism and inhibition potency of verbascoside for cyp enzymes. Molecules.

[29] Akbay P, Calis I, Undeger U, Basaran N, Basaran AA (2002). In vitro immunomodulatory activity of verbascoside from Nepeta ucrainica L. Phytother Res.

[30] Attia YM, El-Kersh DM, Wagdy HA, Elmazar MM (2018). Verbascoside: Identification, quantification, and potential sensitization of colorectal cancer cells to 5-fu by targeting pi3k/akt pathway. Sci Rep.

[31] Alipieva K, Korkina L, Orhan IE, Georgiev MI (2014). Verbascoside--a review of its occurrence, (bio)synthesis and pharmacological significance. Biotechnol Adv.

[32] Paola RD, Oteri G, Mazzon E, Crisafulli C, Galuppo M, Toso RD (2011). Effects of verbascoside, biotechnologically purified by Syringa vulgaris plant cell cultures, in a rodent model of periodontitis. J Pharm Pharmacol.

[33] Mechri B, Tekaya M, Hammami M, Chehab H (2019). Root verbascoside and oleuropein are potential indicators of drought resistance in olive trees (Olea europaea L ). Plant Physiol Biochem.

[34] Yang L, Zhang B, Liu J, Dong Y, Li Y, Li N (2019). Protective effect of acteoside on ovariectomy-induced bone loss in mice. Int J Mol Sci.

[35] Khullar M, Sharma A, Wani A, Sharma N, Sharma N, Chandan BK (2019). Acteoside ameliorates inflammatory responses through NFkB pathway in alcohol induced hepatic damage. Int Immunopharmacol.

[36] Hosseinian S, Shahraki S, Ebrahimzadeh Bideskan A, Shafei MN, Sadeghnia HR, Soukhtanloo M (2019). Thymoquinone alleviates renal interstitial fibrosis and kidney dysfunction in rats with unilateral ureteral obstruction. Phytother Res..

[37] Liu M, Zhu Y, Sun Y, Wen Z, Huang S, Guixia (2018). MnTBAP therapy attenuates the downregulation of sodium transporters in obstructive kidney disease. Oncotarget.

[38] Ma X, Chang Y, Xiong Y, Wang Z, Wang X, Xu Q (2019). Eplerenone ameliorates cell pyroptosis in contralateral kidneys of rats with unilateral ureteral obstruction. Nephron.

[39] Kinter M, Wolstenholme JT, Thornhill BA, Newton EA, McCormick ML, Chevalier RL (1999). Unilateral ureteral obstruction impairs renal antioxidant enzyme activation during sodium depletion. Kidney Int.

[40] Rouschop KM, Sewnath ME, Claessen N, Roelofs JJ, Hoedemaeker I, van der Neut R (2004). CD44 deficiency increases tubular damage but reduces renal fibrosis in obstructive nephropathy. J Am Soc Nephrol.

[41] Singh N, Shukla N, Singh P, Sharma R, Rajendran SM, Maurya R (2010). Verbascoside isolated from Tectona grandis mediates gastric protection in rats via inhibiting proton pump activity. Fitoterapia.

[42] Xia D, Zhang Z, Zhao aY (2018). Acteoside Attenuates oxidative stress and neuronal apoptosis in rats with focal cerebral ischemia - reperfusion injury. Biol Pharm Bull.

[43] Wang L, Ren X, Tian XF, Cheng XL, Zhao YY, Li QY (2019). Protective effects of GPR120 agonist-programmed macrophages on renal interstitial fibrosis in unilateral ureteral obstruction (UUO) rats. Biomed Pharmacother.

[44] Li Y, Liu J, Yu T, Yan B, Li H (2019). Interleukin-33 promotes obstructive renal injury via macrophages. Mol Med Rep.

[45] Wiezel D, Assadi MH, Landau D, Troib A, Kachko L, Rabkin R (2014). Impaired renal growth hormone JAK/STAT5 signaling in chronic kidney disease. Nephrol Dial Transplant.

[46] Yang SX, Zhang ZC, Bai HL (2019). ClC-5 alleviates renal fibrosis in unilateral ureteral obstruction mice. Hum Cell.

[47] Tammaro A, Florquin S, Brok M, Claessen N, Butter LM, Teske GJD (2018). S100A8/A9 promotes parenchymal damage and renal fibrosis in obstructive nephropathy. Clin Exp Immunol.

[48] Liu Y (2011). Cellular and molecular mechanisms of renal fibrosis. Nat Rev Nephrol.

[49] Wang W, Zhou P-H, Hu W, Xu C-G, Zhou X-J, Liang C-Z (2018). Cryptotanshinone hinders renal fibrosis and epithelial transdifferentiation in obstructive nephropathy by inhibiting TGF-β1/Smad3/integrin β1 signal. Oncotarget..

[50] de Moura Sperotto ND, Steffens L, Veríssimo RM, Henn JG, Péres VF, Vianna P (2018). Wound healing and anti-inflammatory activities induced by a Plantago australis hydroethanolic extract standardized in verbascoside. J Ethnopharmacol.

[51] Dimitrova P, Alipieva K, Stojanov K, Milanova V, Georgiev MI (2019). Plant-derived verbascoside and isoverbascoside regulate Toll-like receptor 2 and 4-driven neutrophils priming and activation. Phytomedicine.

[52] Spínola V, Castilho PC (2019). Madeira moneywort (Sibthorpia peregrina L ) as a new source of verbascoside and its derivatives with potential phyto-pharmaceutical applications. Nat Prod Res.

[53] Jia WQ, Wang ZT, Zou MM, Lin JH, Li YH, Zhang L (2018). Verbascoside inhibits glioblastoma cell proliferation, migration and invasion while promoting apoptosis through upregulation of protein tyrosine phosphatase shp-1 and inhibition of stat3 phosphorylation. Cell Physiol Biochem.

[54] Li M, Zhou F, Xu T, Song H, Lu B (2018). Acteoside protects against 6-OHDA-induced dopaminergic neuron damage via Nrf2-ARE signaling pathway. Food Chem Toxicol.

[55] Nishida M, Okumura Y, Fujimoto S-i, Shiraishi I, Itoi T, Hamaoka K (2005). Adoptive transfer of macrophages ameliorates renal fibrosis in mice. Biochem Biophys Res Commun.

[56] Nan Si, Hajime Kanazawa, Katsuki Okuyama, Keisuke Imada, Hongjie Wang bJY, Haiyu Zhao (2018). Involvement of catechols in acteoside in the activation of promatrix metalloproteinase-2 and membrane type-1-matrix metalloproteinase expression via a phosphatidylinositol-3-kinase pathway in human dermal fibroblasts. Biol Pharm Bull..

[57] Alston CI, Dix RD (2019). SOCS and herpesviruses, with emphasis on cytomegalovirus retinitis. Front Immunol.

[58] Illanueva EC, Myers MG Jr (2008). Leptin receptor signaling and the regulation of mammalian physiology. Int J Obes (Lond).

